# The complete mitochondrial genome of the endemic Iberian pygmy skate *Neoraja iberica* Stehmann, Séret, Costa, & Baro 2008 (Elasmobranchii, Rajidae)

**DOI:** 10.1080/23802359.2021.1884030

**Published:** 2021-03-15

**Authors:** André Gomes-dos-Santos, André M. Machado, Sofia Graça Aranha, Ester Dias, Ana Veríssimo, L. Filipe C. Castro, Elsa Froufe

**Affiliations:** a CIIMAR/CIMAR – Interdisciplinary Centre of Marine and Environmental Research, Universityof Porto, Matosinhos, Portugal; b Department of Biology, Faculty of Sciences, University of Porto, Porto, Portugal; c CCMAR – Centre of Marine Sciences, Universidade do Algarve, Faro, Portugal; d CIBIO – Research Centre in Biodiversity and Genetic Resources, Vairão, Portugal

**Keywords:** Chondrichthyes, Elasmobranchii, Iberian Peninsula, mitogenome, phylogenetics

## Abstract

Skates, Chondrichthyes fishes from order Rajiformes, are the most species-rich group of all Batoidea. However, their phylogenetic relationships and systematics is still a highly discussed and controversial subject. The use of complete mitogenome has shown to be a promising tool to fill this gap of knowledge. Here, the complete mitogenome of the Iberian pygmy skate *Neoraja iberica* (Stehmann, Séret, Costa & Baro 2008) was sequenced and assembled. The mitogenome is 16,723 bp long and its gene content (i.e. 13 protein-coding genes, 22 transfer RNA, and 2 ribosomal RNA genes) and arrangement are the expected for Batoidea. Phylogenetic reconstructions, including 89 Rajiformes and two outgroup Rhinopristiformes, recovered family Rajidae as monophyletic, and further divided in the monophyletic tribe Rajini, sister to tribes Amblyrajini and Rostrorajini. The newly sequenced *N. iberica* mitogenome is the first representative of the tribe Rostrorajini.

Within the Batoidea (skates, stingrays, sawfishes, electric rays, and guitarfishes), the order Rajiformes (skates) is one of the most species rich (250 species so far described) despite its seemingly morphological stasis (Ebert and Compagno 2008). Skates also tend to have very localized distributions, reflected in a high degree of endemism with several new species being described recently (Iglésias et al. 2010; Stehmann et al. 2008; Stevenson et al. 2004). Nevertheless, phylogenetic relationship inferences and systematics of the Batoidea remain controversial mostly due to low taxon sampling, unresolved/poorly supported topologies using either morphological and molecular data, and also incongruent morphological and molecular topologies (Aschliman, 2011; Aschliman et al. 2012; Douady et al. 2003; Gaitán-Espitia et al. 2016; Last, White, et al. 2016; McEachran and Aschliman 2004; Rodríguez-Cabello et al. 2013; Serra-Pereira et al. 2011). Although the application of molecular approaches have been fundamental to understand the phylogenetic relationships and assert the systematics within Rajiformes, these are still under discussion with several interpretations available (Last, Weigmann, et al. 2016; Last, White, et al. 2016). Consequently, from here forward, we follow the nomenclature proposed by the most recent taxonomic/systematic review of Rajiformes (Last, Weigmann, et al. 2016).

Skates (Order Rajiformes) are among the most threatened groups of vertebrates and are particularly prone to overexploitation (Davidson et al. 2016; Dulvy et al. 2014). Thus, the inability to efficiently infer phylogenetic relationships and assert the systematics of the group is clearly a concern for fishing management and conservation planning. Most species have low fecundity, late sexual maturity, and long generation times that hinder efficient population restocking and since skates’ meat and gill rakers are valuable commercial resources, over-fishing has devastating effects in many populations (Serra-Pereira et al. 2011; Wannell et al. 2020). This is further aggravated by frequently bottom trawling bycatch of these organisms (Wannell et al. 2020). Complete mitogenomes have been successfully used for comparative studies and phylogenetic inferences in cartilaginous fishes (e.g. Alam et al. 2014; Gaitán-Espitia et al. 2016; Gomes-dos-Santos et al. 2020; Inoue et al. 2010). This is especially relevant in cartilaginous fish due to their slow mtDNA mutation rates that can hinder the efficient resolution of traditional partial mitochondrial markers (Gaitán-Espitia et al. 2016; Martin 1995).

The Iberian pygmy skate *Neoraja iberica* (Stehmann et al. 2008) was recently characterized as an endemic species from the south coast of Portugal and Spain (Serra-Pereira et al. 2011; Stehmann et al. 2008). The very few studies published to date on this species relied on morphological analyses, with only a few partial COI mitochondrial sequences being available. Furthermore, no mitogenomes for *Neoraja* genus are currently available. Therefore, producing a complete mitogenome is a timely and valuable resource.

Liver and muscle tissue samples from a *N. iberica* specimen were collected and stored in 96% ethanol. The specimen was captured in June 2020 off the south coast of Portugal (between Lat: 36.777.215, Long: −8.817.514 and Lat: 36.779.223, Long: −8.612.814), onboard of a commercial crustacean bottom trawler and at depths of approximately 500 m. Morphological identification was performed onboard. The specimen is stored at the Interdisciplinary Center of Marine and Environmental Research (specimen code Neoib001). Liver tissue was used for genomic DNA extraction and whole-genome sequencing of 150 bp paired-end (PE) reads were obtained using Hiseq X Ten machine at Novogene Europe.

Complete mitogenome assembly and annotation were obtained using MitoZ version 2.3. (Meng et al. 2019). Annotation was further validated by comparison with mitogenomes from other members of the Family Rajidae available on NCBI, including representatives of the two rajid tribes: Amblyrajini and Rajini. For the phylogenetic analysis, all available mitogenomes from species from Family Rajidae and from two species from Order Rhinopristiformes (Accession numbers NC_023951.1 and NC_022821.1) were retrieved from GenBank (03/12/2020). The 13 protein-coding genes (PCGs) were individually aligned using MAFFT version 7.453 (Katoh and Standley 2013) and afterward concatenated using FASconCAT-G (https://github.com/PatrickKueck/FASconCAT-G) (final length: 11,435 bp). The best partition-scheme, best fitted evolutionary models and maximum-ikelihood (ML) phylogeny were obtained using IQ-TREE version 1.6.12 (Kalyaanamoorthy et al. 2017; Nguyen et al. 2015). The newly sequenced complete mitogenome of *N. iberica* is available in GenBank under the accession number MW377218. The length of the mitogenome is 16,723 bp and the gene composition and arrangement is, as expected for Batoidea, the typical for vertebrate mtDNA: 13 PCGs, 22 transfer RNA, 2 ribosomal RNA genes, with 14 tRNA, 2 rRNA all PCG (except NAD6) being present in the heavy strand (Satoh et al. 2016).

The resulting phylogenetic tree (Figure 1) recovered Family Rajidae as monophyletic, further divided in the monophyletic tribe Rajini, sister to tribes Amblyrajini and Rostrorajini. The newly sequenced *N. iberica* represents the first mitogenome sequenced Rostrorajini taxa. The present study highlights the importance of increasing the sampling and mitogenome sequencing of Rostrorajini, as well as other skates, to clarify the phylogenetic relationships within the most species-rich group of Chondrichthyes.

**Figure 1.**
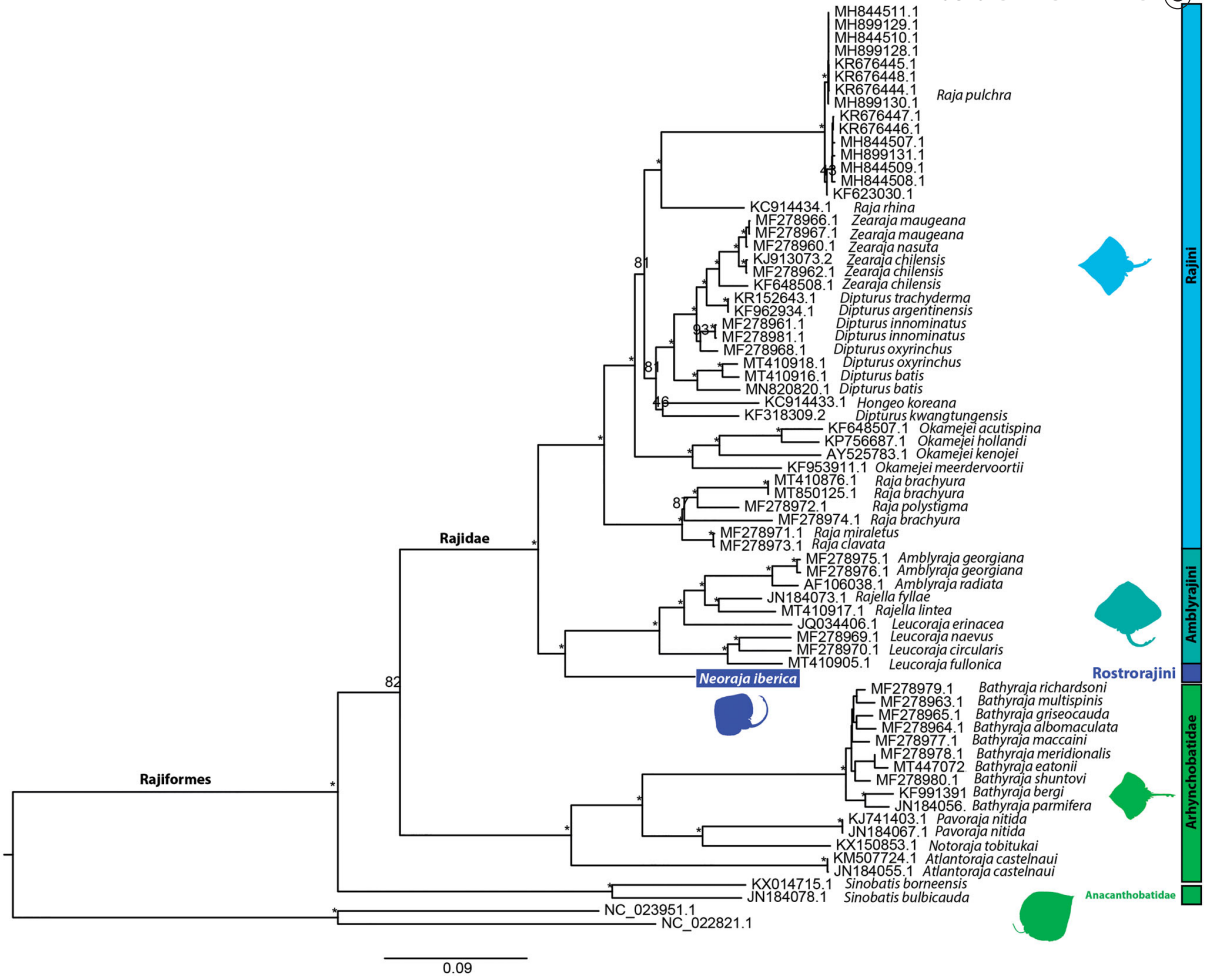
Maximum likelihood phylogenetic tree based on concatenated sequences of 13 protein-coding genes from 89 Rajiformes and two outgroup Rhinopristiformes mitogenomes. GenBank accession numbers are presented before species names. The * above the branches indicate both bootstrap support values above 95%.

## Data Availability

The genome sequence data that support the findings of this study are openly available in GenBank of NCBI at (https://www.ncbi.nlm.nih.gov/) under the accession number MW377218. The associated BioProject, SRA, and Bio-Sample numbers are PRJNA694536, SRS8105111, and SAMN17526303, respectively.
